# Pathology associated with *Tripaphylus* Richiardi, 1878 infection (Copepoda: Sphyriidae) in wild-caught Australian blackspot sharks, *Carcharhinus coatesi* (Whitley, 1939), off northern Australia

**DOI:** 10.1007/s00436-022-07662-w

**Published:** 2022-09-12

**Authors:** Diane P. Barton, Amy Kirke, Grant Johnson, Geoff Boxshall

**Affiliations:** 1 School of Agricultural, Environmental and Veterinary Sciences, Charles Sturt University, Wagga Wagga, NSW 2678 Australia; 2 Research Institute of Environment and Livelihood, Charles Darwin University, Darwin, NT 0801 Australia; 3 Fisheries Research Division, Northern Territory Department of Industry, Tourism and Trade, Darwin, NT 0801 Australia; 4 Department of Life Sciences, Natural History Museum, Cromwell Road, London, SW7 5BD UK

**Keywords:** Elasmobranch, Histopathology, Marine, Inflammation, Host-parasite interactions

## Abstract

Female specimens of the newly described mesoparasitic copepod *Tripaphylus squidwardi* (Sphyriidae), collected from the Australian blackspot shark, *Carcharhinus coatesi*, off northern Australia were examined histologically. The ‘encapsulated’ head of the copepod was found in the ventral musculature of the throat of the shark. The head of the copepod was surrounded by a tissue capsule of unknown origin. There were signs of chronic inflammation associated with the infection, although there appeared to be no effect on the health of the shark.

Wild-caught specimens of *Carcharhinus coatesi* (Whitley, 1939) were examined for parasites as part of an ongoing project exploring their biology and ecology. A total of 233 *C. coatesi* was collected from 13 May 2018 to 8 Nov 2019 as bycatch within the Demersal and Timor Reef Fisheries which operate within the Northern Territory exclusive economic zone, in northern Australia. Sharks were either frozen or refrigerated whole after capture on the fishing boats; once onshore, all sharks were then stored frozen until processing.

At the time of processing, the head of the shark was removed posterior to the gill region. The gills were then individually examined for the presence of the posterior trunk of female *Tripaphylus* Richiardi, 1878 (Fig. 1A). When found, attempts were made to dissect the neck and head of the copepod from the shark specimen (Fig. 1C). In total, 46 specimens of *Tripaphylus* were collected from 27 *C. coatesi* (prevalence of infection 11.6%, mean intensity of infection 1.7, range of infection 1–6). Eleven complete females were collected, while the remaining specimens were damaged or missing the anterior end. These specimens were identified as belonging to a recently described new species, *T. squidwardi* Boxshall et al. 2022 (Boxshall et al. 2022).

**Fig. 1:**
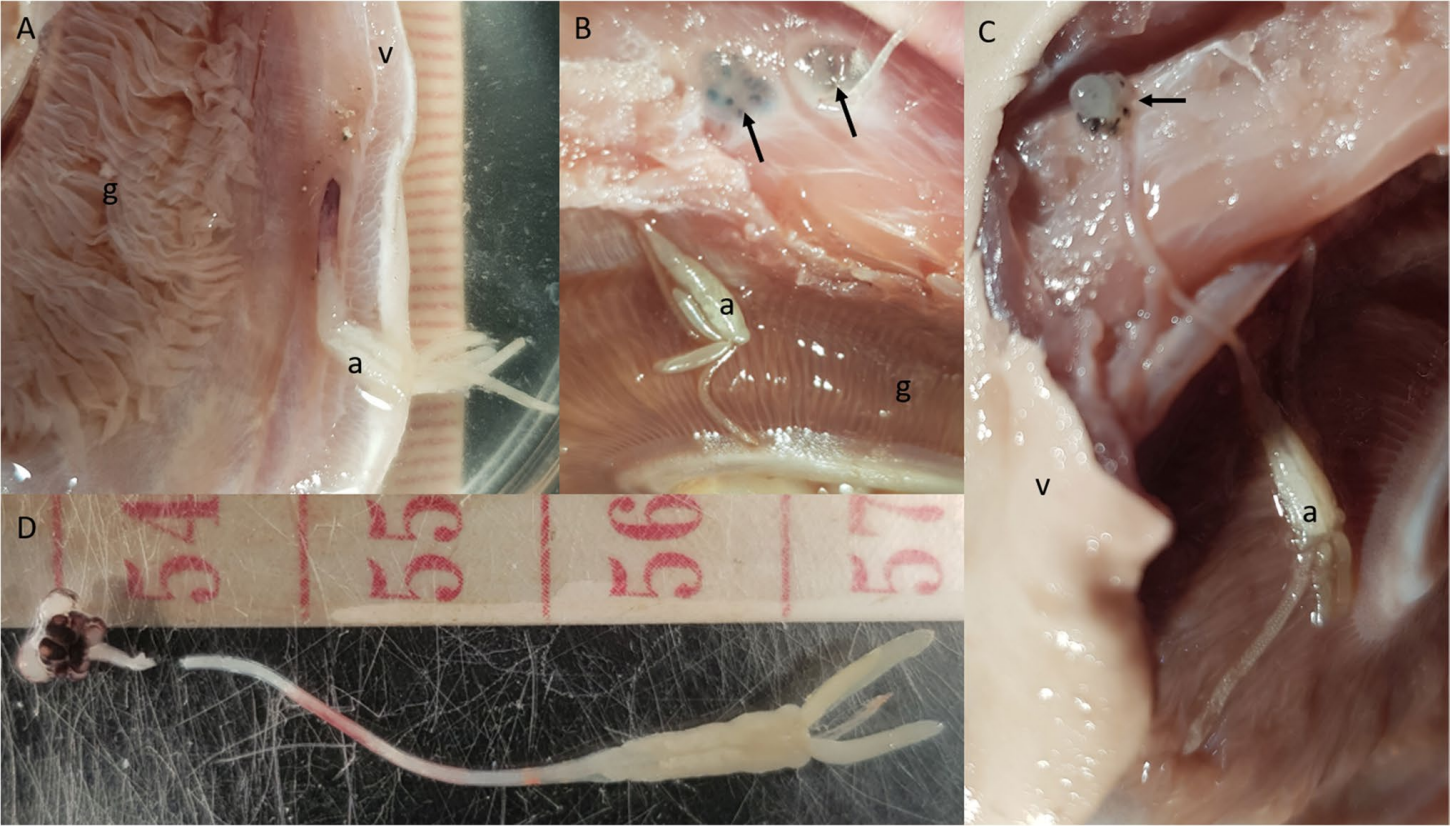
Female specimen of *Tripaphylus squidwardi* collected from *Carcharhinus coatesi*. **A** Female embedded into the ventral gill flap with the abdomen visible in region of the gill. Tape measure indicates millimetres. **B** Ventral skin removed from the throat region of the shark, exposing the head (arrow) of two specimens of *Tripaphylus squidwardi*. **C** A female *T. squidwardi* fully dissected from the ventral throat region of the shark in situ. **D** A female *T. squidwardi* removed from the shark. Tape measure indicates centimetres. a, abdomen of copepod; g, gill of shark; v, ventral surface of shark

Two heads of *T. squidwardi* embedded in the ventral throat musculature of *C. coatesi* were collected in situ and preserved in 10% buffered formalin for histopathology. The samples were submitted to the Charles Sturt University Veterinary Diagnostic Laboratory for processing and histological assessment. The samples were embedded in paraffin blocks. Sections were cut at 4 µm and placed onto positively charged slides before being heated at 60 °C for 20 min. The sections were stained with haematoxylin and eosin using a Leica autostainer and then coverslipped with Pertex mountant before drying. The slide material has been deposited in the collection of the Museum and Art Gallery of the Northern Territory (NTM Cr019500).

During the dissection of the specimens of *T. squidwardi* from the tissues of the shark, it was noted that the neck was contained within a ‘tube’ of tissue (the dark pink colouration of the neck region as it enters the host tissue in Fig. 1A). The head of the copepod was also ‘encapsulated’ within the musculature of the host.

Due to the initial freezing of the specimens, moderate to advanced autolysis interfered with histological interpretation. However, examination of the sections showed areas of inflammation surrounding the embedded head of the copepod (Fig. 2). Infiltration of mononuclear cells was evident within the area of inflammation.

**Fig. 2:**
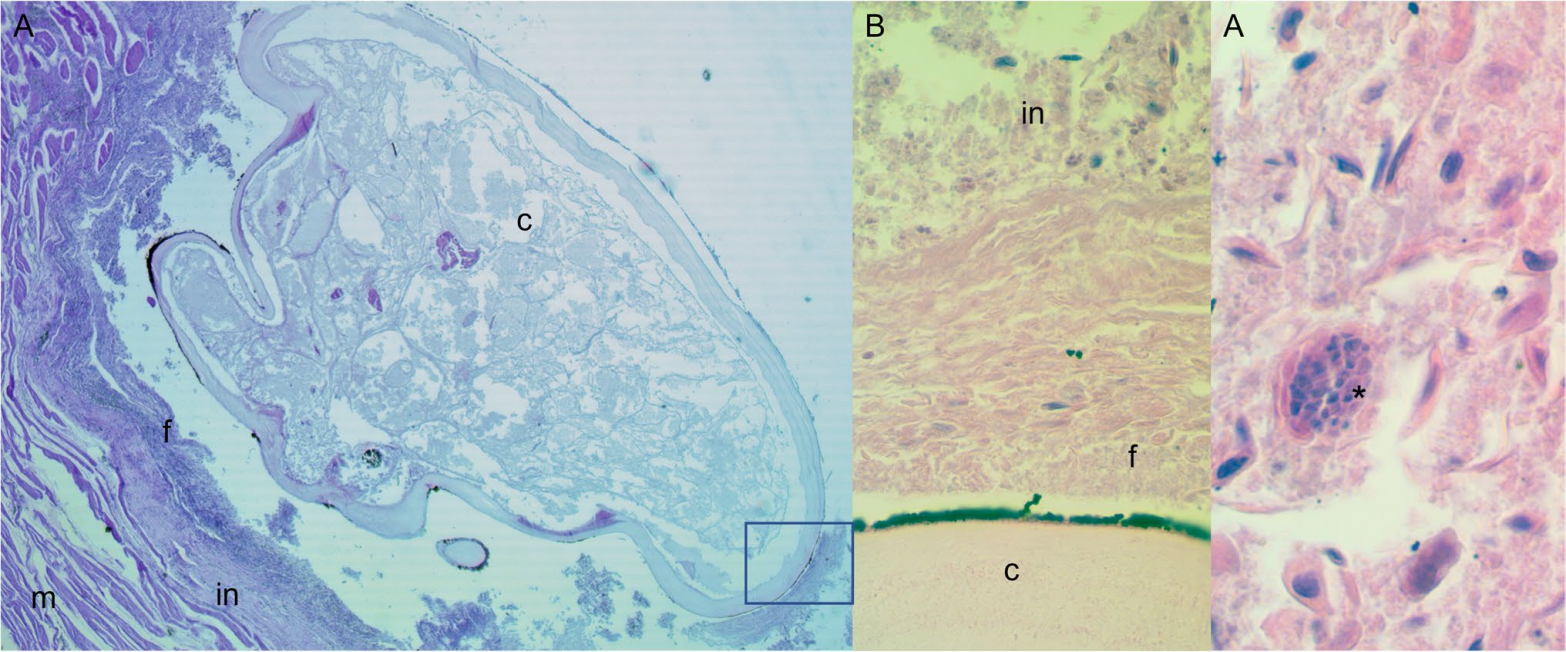
Histological section of embedded head of female specimen of *Tripaphylus squidwardi* in the ventral throat musculature of *Carcharhinus coatesi*. **A** Whole head surrounded by capsule and inflammation. Square indicates section in **B**. **B** Higher magnification of area of capsule and inflammation surrounding the embedded head. **C** Mononuclear cell (*) within area of inflammation. c, head of copepod; f, fibrotic capsule surrounding head; in, area of inflammation; m, musculature of host

Although inflammation was noted at the site of the embedded head, there appears to be no obvious effect on the health of the shark, as infected sharks appeared in good body condition, with livers of normal appearance. The location of the embedded head was not obvious externally but was observable as a dark spotted area once the ventral skin was removed (Fig. 1B). The ventral side of the copepod head was often surrounded by extravasated blood which typically formed dark clots on the surface of the head lobes and limbs. The presence of clots adhering to the limbs makes observation of their detailed setation problematic.

Although there have been studies of the pathological impact of species of *Sphyrion* Cuvier, 1830 on their teleost hosts (see Erlingsdóttir et al. 2020 for a recent example), there have been no previous studies on the pathology associated with sphyriid copepods infecting elasmobranchs. Site of infection by *Sphyrion lumpi* (Krøyer, 1845) in beaked redfish, *Sebastes mentella* Travin, 1951, had evidence of considerable infiltration of leucocytes surrounding a host-produced layer of fibrous tissues around the embedded head and neck of the copepod (Erlingsdóttir et al. 2020). The area of inflammation evident in the samples examined in this study was not as pronounced, although mononuclear cells were present. Additionally, unlike Erlingsdóttir et al. (2020), there was no obvious degeneration of the surrounding muscle cells. It is possible that the capsule encasing the *T. squidwardi* is also produced by the host, however, this was unable to be determined.

*Tripaphylus* spp. are mesoparasitic crustaceans, with the head embedded into the tissues of the host, and the female trunk and/or eggs sacs protruding into the gill slits (Benz 1993; MacKenzie and Smith 2016; this study). As such, this parasite is permanently fixed to the host (Benz 1993). Captive specimens of *Mustelus asterias* Cloquet, 1819 in Scotland, infected with *Tripaphylus musteli* (van Beneden, 1851), all appeared healthy and were feeding well (MacKenzie and Smith 2016). However, the understanding of elasmobranch physiology and the effects of parasites, such as these copepods, remains understudied (Borucinska et al. 2020; Johnson et al. 2019).

## Data Availability

Material has been deposited in a curated museum collection and data will be made available upon reasonable request.
